# Gastric peroral endoscopic pyloromyotomy as an effective treatment for symptomatic delayed gastric emptying after Ivor Lewis esophagectomy

**DOI:** 10.1016/j.igie.2025.09.005

**Published:** 2025-09-11

**Authors:** Tarek Nammour, Makoto Nishimura, Natalia Zbib, Seung Eun Lee, Sayaka Nagao, Mako Koseki, Daniela Molena, David R. Jones, Hans Gerdes, Jacques Beauvais, Mark Schattner

**Affiliations:** 1 Division of Gastroenterology and Hepatology, Department of Internal Medicine, Saint Louis University Hospital, St. Louis, Missouri, USA; 2 Gastroenterology, Hepatology, and Nutrition Service, Department of Medicine, Memorial Sloan Kettering Cancer Center, New York, New York, USA

## Abstract

**Background and Aims:**

Delayed gastric emptying (DGE) is a frequent adverse event after Ivor Lewis esophagectomy, negatively affecting quality of life. Although gastric peroral endoscopic pyloromyotomy (G-POEM) is effective for gastroparesis, its role in postesophagectomy DGE remains uncertain. This study evaluates the feasibility and outcomes of G-POEM in this setting.

**Methods:**

We conducted a single-center retrospective analysis of 11 patients who underwent G-POEM after Ivor Lewis esophagectomy, all of whom had symptomatic DGE confirmed by gastric-emptying studies and Gastroparesis Cardinal Symptom Index scores.

**Results:**

All patients had confirmed DGE, with a median 49% (interquartile range, 24-80) gastric retention at 4 hours on gastric-emptying studies (normal is ≤10% retention at 4 hours). After G-POEM, gastric emptying normalized in 50% of patients and improved in 37.5%, with a median retention of 13% (0-75) at 4 hours. The median Gastroparesis Cardinal Symptom Index score improved from 25 (11-33) to 11 (2-33), where a score of 0 indicates no symptoms. Prokinetic use declined from 45.5% to 9.1%. The median procedure time was 68 (33-110) minutes, hospital stay was 1 (1-5) day, and no immediate adverse events were reported.

**Conclusions:**

G-POEM appears to be a feasible and safe treatment for DGE after Ivor Lewis esophagectomy, associated with early symptomatic improvement and minimal hospital stay although further prospective studies are needed.

## Introduction

Esophagectomy is considered the reference standard for the surgical management of esophageal cancer. However, this surgery is associated with significant morbidity and mortality. The incidence of delayed gastric emptying (DGE) postesophagectomy ranges between 10% and 50%.^1^ This is likely because of the necessary vagotomy performed during lymphadenectomy and gastric transection, which disrupts the parasympathetic innervation of the stomach. DGE can severely impact patients' quality of life, leading to persistent nausea, difficulties in oral intake, weight loss, and adverse events such as recurrent aspiration pneumonia.

Gastric peroral endoscopic pyloromyotomy (G-POEM) has emerged as a potentially effective treatment modality for medically refractory gastroparesis. It is generally considered a safe procedure with high technical success rates, especially when performed by an endoscopist skilled in third-space endoscopy. Although the long-term clinical success rate of G-POEM falls within the 50% to 60% range, it still provides significant clinical benefits for patients with refractory gastroparesis.^2^ To our knowledge, no previous studies have examined the role of G-POEM in patients postesophagectomy. This study aims to assess the effectiveness of G-POEM in patients experiencing DGE symptoms after Ivor Lewis esophagectomy.

## Methods

We conducted a retrospective study at a large tertiary cancer center involving patients who underwent Ivor Lewis surgery for esophageal cancer and subsequently received G-POEM for the management of DGE symptoms between November 2021 and May 2023. All patients who developed symptomatic DGE after Ivor Lewis esophagectomy and were referred for the G-POEM procedure were included (n = 11). All G-POEM procedures were performed by a single expert endoscopist, beginning with a mucosal entry incision at the level of the gastric antrum to create access to the submucosal space. This was followed by submucosal tunneling toward the pylorus. A pyloromyotomy was then performed by selectively cutting the circular muscle fibers of the pyloric sphincter (single myotomy). Finally, the mucosal entry was closed using endoscopic clips. The endoluminal functional lumen imaging probe (ie, EndoFLIP; Medtronic, Minneapolis, Minn, USA) is an imaging tool used to assess the biomechanical properties of the pylorus. It has been used during G-POEM procedures to evaluate pyloric distensibility, guide myotomy treatment, and predict clinical outcomes. However, this device is not available at our institution and was therefore not used to evaluate G-POEM outcomes in our study. The study was approved by the institutional review board (Memorial Sloan Kettering Cancer Center 20-495 A). The primary outcome was treatment effectiveness, evaluated using the Gastroparesis Cardinal Symptom Index (GCSI) and gastric-emptying studies both before the G-POEM procedure and at the 3-month follow-up. Secondary outcomes included the need for prokinetics, procedure-related adverse events, and length of hospital stay. Data are presented as median (interquartile range [IQR]) unless otherwise specified. All analyses were performed using IBM SPSS Statistics for Windows, Version 24.0 (IBM Corp, Armonk, NY, USA).

## Results

Between November 2021 and May 2023, a total of 11 G-POEM procedures were performed for symptomatic DGE after Ivor Lewis esophagectomy (Table 1). The median age of the patients was 63 years (IQR, 53-77), with a female prevalence of 54.5%. The median Charlson Comorbidity Index was 4 (3-7). The majority of patients (90.9%) had a history of esophageal adenocarcinoma, and all were in cancer remission at the time of the G-POEM. The median time between Ivor Lewis surgery and referral to the gastroenterology office was 64 months (15-90), and the median time between Ivor Lewis surgery and the G-POEM procedure was approximately 74 months (48-93). Pretreatment evaluation revealed that the majority of patients (81.8%) had previously undergone both botulinum toxin injection into the pylorus and endoscopic dilation via through-the-scope balloon dilators up to 20 mm. None of the patients underwent surgical myotomy at the time of the Ivor Lewis procedure or were on opioids, and all G-POEM procedures were performed using a single myotomy technique.

**Table 1:** Baseline characteristics

Characteristics	Data
Number of patients	11
Age, years, median (IQR)	63 (53-77)
Sex, n (%)	6 (54.5%)
Charlson Comorbidity Index, median (IQR)	4 (3-7)
Esophageal cancer type, n (%)	
Adenocarcinoma	10 (90.9%)
Squamous cell carcinoma	1 (9.1%)
Cancer in remission, n (%)	11 (100%)
Previous botulinum toxin injection treatment, n (%)	9 (81.8%)
Previous endoscopic dilation, n (%)	9 (81.8%)

*IQR*, Interquartile range.

Table 2 summarizes the primary and secondary outcomes. The median GCSI score was 25 (11-33) before treatment, indicating significant symptomatology (a score of 0 indicates no symptoms). Gastric-emptying studies revealed 100% DGE in the cohort, with a median of 49% (24%-80%) retained food after 4 hours (normal result is ≤10% gastric retention at 4 hours). Three months post-G-POEM, the median GCSI improved to 11 (2-33), indicating a significant reduction in symptoms. Gastric-emptying studies showed normalization in 50% of patients, improvement in 37.5%, and worsening of radiologic findings with an increased percentage of retained food in 12.5% despite significant improvement in GCSI scores. Posttreatment, the median percentage of retained food after 4 hours was reduced to 13% (0-75). Prokinetic use decreased from 45.5% to 9.1% after G-POEM. The median endoscopic time was 68 minutes (33-110) with no reported immediate adverse events. The median postprocedure hospital stay was 1 day (1-5). One patient required percutaneous endoscopic jejunostomy after G-POEM for refractory symptomatic DGE.

**Table 2:** Primary and secondary outcomes

	Before G-POEM	After G-POEM	*P* value
GCSI, median (IQR)	25 (11-33)	11 (2-33)	.004
Gastric-emptying study, n (%)∗			.002
Delayed	8 (100%)	1 (12.5%)	
Normal	0 (0%)	4 (50%)	
Improved		3 (37.5%)	
% retained food after 4 hours, median (IQR)	49% (24-80)	13% (0-75)	.075
On prokinetics, n (%)	5 (45.5%)	1 (9.1%)	.045
G-POEM procedure time, minutes, median (IQR)	68 (33-110)		
Adverse events, n (%)	0		
Length of hospital stay, days, median (IQR)	1 (1-5)		
Requiring PEJ after G-POEM, n (%)	1 (9.1%)		

*GCSI*, Gastroparesis Cardinal Symptom Index; *G-POEM*, gastric peroral endoscopic pyloromyotomy; *IQR*, interquartile range; *PEJ*, percutaneous endoscopic jejunostomy.

∗ Missing data on 3 patients.

## Discussion

This study suggests that G-POEM is a promising therapeutic option for the management of symptomatic DGE in patients after Ivor Lewis esophagectomy. The significant improvement in GCSI scores and the reduction in the percentage of retained food observed in this cohort shortly after G-POEM underscore the potential of this procedure to alleviate the debilitating symptoms associated with postesophagectomy DGE. Notably, 50% of the patients demonstrated normalized gastric-emptying after treatment, with an additional 37.5% showing improvement, highlighting the procedure's effectiveness even in this challenging patient population. Given that most patients had undergone previous interventions, including botulinum toxin injection treatment and endoscopic dilation, the observed clinical improvements post-G-POEM suggest that this procedure may offer benefits where other treatments have failed. In addition, the reduction in the use of prokinetics post-G-POEM further supports its role in managing refractory cases, potentially reducing the need for ongoing pharmacologic treatment. Endoscopic balloon dilation has been shown to be an effective and safe treatment option for refractory gastroparesis; however, patients often require multiple sessions.^3^ Intrapyloric botulinum toxin injection demonstrated effectiveness in open-label trials, although randomized trials have not confirmed its superiority over placebo.^4^ Interestingly, a recent study found that the response to dilation or botulinum toxin was not a predictor of response to G-POEM. However, patients who had previously received botulinum toxin were more likely to experience postoperative improvement.^5^

Gastric-emptying studies have traditionally been the primary diagnostic tool for gastroparesis because of their cost-effectiveness and accessibility. However, there remains a lack of universally accepted criteria and standardized tools for diagnosing and assessing DGE severity in patients postesophagectomy. Recently, an international expert consensus proposed diagnostic criteria for early and late DGE in these patients, using a symptom grading tool.^6^ In this study, we incorporated both a symptom-scoring questionnaire and radiologic gastric-emptying tests to evaluate G-POEM's efficacy. The GCSI score was developed and validated to assess gastroparesis symptom severity, although not specifically for patients postesophagectomy. However, the GCSI scoring system incorporates recommended questions from the newly established international consensus and has been used in previous studies and randomized trials to evaluate the outcomes of endoscopic pyloromyotomy for treating gastroparesis. The American Gastroenterological Association Clinical Practice Guidelines also recommend that the GCSI score be recorded at each clinic visit following G-POEM.^7^

The absence of immediate adverse events and the relatively short hospital stay postprocedure indicate that G-POEM is not only effective but also safe and well-tolerated. A systematic review and meta-analysis evaluating the long-term outcomes (≥3 years) of G-POEM found that it provides durable clinical success with low rates of adverse events.^8^ However, the need for percutaneous endoscopic jejunostomy in 1 patient underscores the importance of careful long-term follow-up. These findings align with the existing literature on G-POEM's use in gastroparesis, where long-term success rates can vary, suggesting that although G-POEM offers substantial benefits, patient selection and postprocedural monitoring are crucial.

One of the strengths of this study is its focus on a specific and under-researched patient population—those experiencing DGE after Ivor Lewis esophagectomy.^9^^,^^10^ To the best of our knowledge, this is the first study to assess the role of G-POEM in this patient population. By evaluating the effectiveness of G-POEM, the study offers important insights into a specialized area of clinical practice in which treatment options are scarce. Furthermore, the study's incorporation of both subjective (GCSI scores) and objective (gastric-emptying studies) metrics provides a comprehensive evaluation of the procedure's impact on patient outcomes, while acknowledging the limitation that these tests are not validated in this specific patient population. The findings related to secondary outcomes, such as the decreased reliance on prokinetics and the absence of immediate adverse events, further strengthen the evidence supporting the safety and effectiveness of G-POEM.

However, the study also has several limitations. The small sample size limits the generalizability of the findings and the statistical power to detect less-common outcomes or adverse events. In addition, the retrospective design of the study may introduce biases that could affect the accuracy of the data collected. The short follow-up period of 3 months also restricts the ability to assess the long-term durability of G-POEM in this patient population. Finally, the study's single-center design may limit the applicability of the results to other clinical settings, where variations in patient populations, endoscopic techniques, and postoperative care might influence outcomes.

## Conclusions

In conclusion, G-POEM appears to be a feasible and safe treatment modality for symptomatic DGE in patients after Ivor Lewis surgery for esophageal cancer. It is associated with early symptom improvement and enhanced gastric emptying, with minimal hospital stay and no immediate adverse events observed. Further studies with larger cohorts and longer follow-up periods are warranted to better define the long-term outcomes and optimize patient selection for this procedure.

## Patient Consent

This article does not discuss individual patients, so no consent was needed.

## Disclosure

The following authors disclosed financial relationships: M. Nishimura: Consultant for Boston Scientific, Olympus America, Fujifilm, Noah Medical, and AI Medical Service Inc. All other authors disclosed no financial relationships.
